# miR‐152‐5p suppresses glioma progression and tumorigenesis and potentiates temozolomide sensitivity by targeting FBXL7

**DOI:** 10.1111/jcmm.15114

**Published:** 2020-03-09

**Authors:** Shiqi Kong, Yanwei Fang, Bingqian Wang, Yingxiao Cao, Runzhi He, Zongmao Zhao

**Affiliations:** ^1^ Department of Neurosurgery The Second Hospital of Hebei Medical University Shijiazhuang Hebei China; ^2^ Department of Neurosurgery Xingtai People's Hospital Xingtai Hebei China

**Keywords:** cancer growth, FBXL7, glioma, miR‐152‐5p, temozolomide

## Abstract

A generally used chemotherapeutic drug for glioma, a frequently diagnosed brain tumour, is temozolomide (TMZ). Our study investigated the activity of FBXL7 and miR‐152‐5p in glioma. Levels of microRNA‐152‐5p (miR‐152‐5p) and the transcript and protein of FBXL7 were assessed by real‐time PCR and Western blotting, respectively. The migratory and invasive properties of cells were measured by Transwell migration and invasion assay and their viability were examined using CCK‐8 assay. Further, the putative interaction between FBXL7 and miR‐152‐5p were analysed bioinformatically and by luciferase assay. The activities of FBXL7, TMZ and miR‐152‐5p were analysed in vivo singly or in combination, on mouse xenografts, in glioma tumorigenesis. The expression of FBXL7 in glioma tissue is significantly up‐regulated, which is related to the poor prognosis and the grade of glioma. TMZ‐induced cytotoxicity, proliferation, migration and invasion in glioma cells were impeded by the knock‐down of FBXL7 or overexpressed miR‐152‐5p. Furthermore, the expression of miR‐152‐5p reduced remarkably in glioma cells and it exerted its activity through targeted FBXL7. Overexpression of miR‐152‐5p and knock‐down of FBXL7 in glioma xenograft models enhanced TMZ‐mediated anti‐tumour effect and impeded tumour growth. Thus, the miR‐152‐5p suppressed the progression of glioma and associated tumorigenesis, targeted FBXL7 and increased the effect of TMZ‐induced cytotoxicity in glioma cells, further enhancing our knowledge of FBXL7 activity in glioma.

## 1 INTRODUCTION

A common form of brain tumour, glioma, accounts for nearly 80% of all malignant tumours and 30% of all brain tumours.^1^, ^2^ Although significant development has been made in the management of glioma, malignant glioma of high grade remains poorly diagnosed with 12‐14 months of median survival.^3^, ^4^ Surgery, radiotherapy and chemotherapy are the primary treatment modes of glioma.^5^, ^6^ Over the past decades, TMZ (temozolomide) has been commonly used as an imidazotetrazine agent effective against glioma, although its clinical application is remarkably limited due to the acquired and inherent resistance of gliomas to TMZ.^7^, ^8^ Therefore, it is crucial to explore new strategies to suppress resistance and enhance responses to TMZ.

F‐box proteins characteristically possess a 40‐amino acid F‐box domain with NH2‐terminal that links to Cul1‐Rbxl by binding to Skp1.^9^ These F‐box proteins are further sub‐categorized as subfamilies L (containing a motif of leucine‐rich repeats), W (containing a motif of WD repeats) and O (F‐box only) based on the carboxyl‐terminal domain.^10^, ^11^ Their WD/leucine‐rich repeat motif enables these proteins to recognize an array of substrates.^12^ The association between F‐box protein and its substrates has facilitated better information on several SCF E3 ligases mediated biological functions, such as inflammation, mitotic cell cycle progression and gene expression.^13^ FBXL7 has been shown to target Aurora kinase A for proteasomal degradation,^14^ and polyubiquitylation, which has a crucial function in formation of mitotic spindles and segregation of chromosome.^15^ Additionally, whereas FBXL7 exhibits pro‐apoptotic activity, the role of FBXL7 on glioma remains to be extensively unravelled.^15^, ^16^

The small (nearly 22 nucleotides long), noncoding, endogenous transcripts, called the microRNAs (miRNAs), participate in translational repression and gene silencing via binding with target mRNAs.^17^, ^18^ Further, miRNAs have been closely associated with progression, initiation, diagnosis, and a prognosis of cancer and cytotoxicity induced by drugs in several cancerous states, such as glioma.^19^, ^20^ As miR‐152 was first discovered in mouse colon in 2002,^21^ more and more studies have shown that miR‐152 is a tumour suppressor that is involved in cell proliferation, invasion and migration of various cancers, including ovarian, gastric and liver carcinomas.^22^ In humans, the miR‐152 gene is located at 17q21.32.^23^ After transcription and nuclear cleavage, the precursor miR‐152 (pre–miR‐152) is transported to the cytoplasm and is cleaved by Dicer into miR‐152 duplex.^24^ Finally, two mature of lengths and sequences were generated from opposite arms of the miR‐152 duplex, called miR‐152‐3p and miR‐152‐5p, respectively.^25^ However, the role of miR‐152‐5p in glioma is not fully understood.

Therefore, in this study, we examined the function of miR‐152‐5p in regulating the growth and progress of tumours by targeting FBXL7 as well as the function of miR‐152‐5p/FBXL7 axis on cytotoxicity induced by TMZ in glioma. We observed that miR‐152‐5p lies upstream of FBXL7 and regulates FBXL7 level and overexpressed miR‐152‐5p repressed glioma development and progression and targets FBXL7 to augment cytotoxicity induced by TMZ in glioma cells.

## 2 MATERIALS AND METHODS

### 2.1 Cell lines and samples of tissues

The tissues samples of glioma and NATs (normal adjacent brain tissues) were obtained from glioma patients at The Second Hospital of Hebei Medical University. The Ethics Committee of The Second Hospital of Hebei Medical University authorized this study. Before our study, each patient gave informed written consent.

NHAs (normal human astrocytes) were procured from Lonza (Switzerland), and H4, A172, LN229, U87 and U251 cell lines were got from ATCC (USA) and cultured using Astrocyte Growth Medium Bullet Kit from Lonza with media for astrocyte growth as well as supplements. Cell lines U87 and U251 were grown in Dulbecco's Modified Eagle's Medium containing foetal bovine serum (FBS, 10%) (Thermo Fisher Scientific).

### 2.2 Plasmid constructs and transfection

The mimic of miR‐152‐5p, miR‐con (negative control), miR‐152‐5p inhibitor and inhibitor‐con were procured from GenePharma Co. Ltd. (China). Subcloning of the coding region of FBXL7 was cloned into pcDNA3.1 plasmid to obtain pcDNA‐FBXL7 (FBXL7) overexpressing construct. TMZ was procured from Selleckchem (USA). Transfection of cell lines was performed with miR‐152‐5p mimic or miR‐152‐5p inhibitor (200 nmol/L each) along with lipofectamine 3000 from Thermo Fisher Scientific as instructed.

### 2.3 Determination of mRNA levels by reverse transcription quantitative PCR assay

Real‐time PCR was performed as previously reported.^26^, ^27^ RNA was isolated using TRIzol reagent from Thermo Fisher Scientific as per instructions supplied. The expression of miR‐152‐5p was performed using MicroRNA Assay kit (Thermo Fisher Scientific) and internal control was RNU6B. RNA was reverse transcribed into cDNA using M‐MLV Reverse Transcriptase from Promega to analyse the expression of FBXL7 and normal control (β‐actin) mRNA and estimated using iTaq™ Universal SYBR® Green Supermix from Bio‐Rad (USA) on the Roche LightCycler 480 system from Roche Diagnostics. The primers used in this study are as follows: hsa‐miR‐152‐5p, Forward: 5′‐ CAGAGGTTCTGTGATACACTC‐3′, Reverse: 5′‐ GGTCCAGTTTTTTTTTTTTTTTAGTC‐3′; U6, Forward: 5′‐CTCGCTTCGGCAGCACA‐3′, U6, Reverse: 5′‐AACGCTTCACGAATTTGCGT‐3′; CSF‐1, Forward: 5’‐ TCTGCACCTTTGTGCTCATC‐3’, Reverse: 5’‐ GCCTCATTAGAGATGTTGTAGC‐3’; β‐actin, Forward: 5′‐CTCACCATGGATGATGATATCGC‐3′, Reverse: 5’‐AGGAAT‐CCTTCTGACCCATGC‐3′.

### 2.4 Western blotting

Western blotting was performed as previously studied.^28^, ^29^ Using RIPA Lysis and Extraction Buffer, glioma cells were lysed in a system containing protease inhibitor mixture. The estimation of protein concentration was estimated utilizing a Pierce BCA Protein Assay Kit (Thermo Fisher Scientific). Then, 50 μg protein in the cell lysates was resolved through SDS‐PAGE and transferred onto PVDF membranes (Millipore). Blocking of the membrane was performed in skimmed milk (5%) and probed at 4°C overnight with primary antibodies (Abcam) against FBXL7 or β‐actin (Abcam). Next, secondary antibody (goat‐anti‐rabbit) and HRP horseradish peroxidase‐conjugated) (Abcam) was added. The obtained bands were visualized with ECL Substrate (Clarity Max Western; Bio‐Rad), and protein bands were densitometrically analysed using Quantity One software from Bio‐Rad. The primary antibodies are as follows: FBXL7 (ab59149, Abcam), Ki‐67 (9449, Cell Signaling technology) and β‐actin (A5441, Sigma).

### 2.5 Reporter assays

FBXL7 3′‐UTR partial wild‐type (WT) sequence harbouring the potential binding site for miR‐152‐5p and its mutant sequences were cloned singly in luciferase vector psiCHECK‐2 and FBXL7 3′‐UTR‐WT and FBXL7 3′‐UTR‐MUT to generate luciferase reporters, respectively. Then, the transfection of either reporter was performed into U87 and U251 cells along with the mimic of miR‐152‐5p or miR‐con (negative control), and the luciferase activity was measured.

### 2.6 Assay for cell invasion and migration

Seeding of cells transfected in a medium free of serum was performed in the upper chamber of Transwell (Corning) Matrigel‐precoated (to assess invasion) or non‐coated (to assess migration). Then, 20% FBS containing medium was plated into chamber sublayers. After incubating for one full day, cotton swabs were used to erase cells on membrane upper surface, and fixing, staining and imaging of cells penetrating the membranes were performed followed by counting them in 15 random fields.

### 2.7 Estimation of cell cytotoxicity and proliferative ability

Assessment of cell cytotoxicity and proliferative ability was examined using the CCK‐8 assay kit from Sigma. In brief, seeding of cells was performed with 96‐well plates. After infection or treatment, 10 μL of CCK‐8 solution was added at specific time‐points per well, kept for 120 min and the optical density was estimated at 450 nm.

### 2.8 Mouse model

Mouse experiments were approved by The Second Hospital of Hebei Medical University's Animal Care and Use Committee and were performed as per animal use and care guidelines by the national standard of laboratory. Fragment of FBXL7 knock‐down in the lentivirus (sh *FBXL7*) and sh con (negative control), and miR‐152‐5p (lenti‐miR‐152‐5p) in lentivirus and its control (lenti‐miR‐con) were constructed. Mice were arbitrarily categorized into groups indicating sh con, TMZ, sh con + TMZ, TMZ + sh *FBXL7*, sh *FBXL7*, lenti‐miR‐con, lenti‐miR‐con + TMZ, TMZ + lenti‐miR‐152‐5p, lenti‐miR‐152‐5p groups and the control group (saline), with six mice per group. U87 cells (Uninfected) or cells (5 × 10^5^) infected with sh *FBXL7*, sh con, lenti‐miR‐152‐5p lentiviruses or lenti‐miR‐con (5 × 10^5^/each) were given administered subcutaneously into the six‐week‐old male nude mice (Balb/c athymic) in its right hind limb. Tumour volume was recorded every 2 days, and the volume of tumours was estimated using a caliper and the formula: volume = ½ × length × width^2^. Seven days later, intraperitoneal administration of TMZ (20 mg/kg) was performed into mice each day for 10 days post‐cell injection. Nineteen days after injection, excision and weighing of tumours were performed.

### 2.9 Statistical analysis

Each experiment was repeated thrice and expressed as means ± SD Analyses of data were performed using GraphPad Prism (USA). Analysis of statistical difference was performed using one‐way ANOVA along with Tukey's post hoc test or Student's t test, keeping *P* < .05 as statistically significant.

## 3 RESULTS

### 3.1 The expression of FBXL7 was significantly enhanced in glioma cells and correlated with the grade of glioma and patient survival

**Figure 1:**
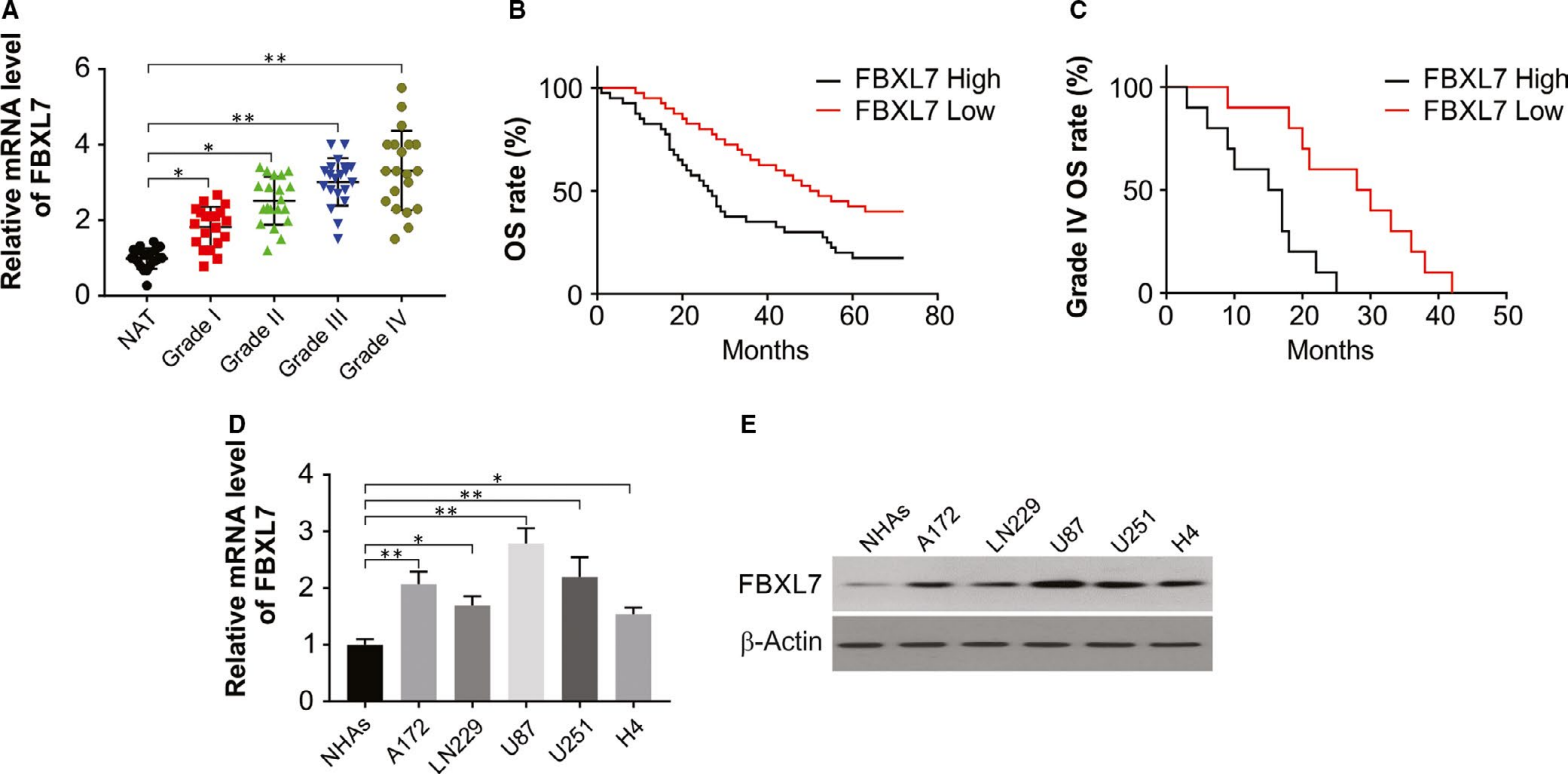
FBXL7 level was up‐regulated in glioma tissues and cells, and related with grade and poor prognosis of glioma. (A) Real‐time PCR was used to detect FBXL7 mRNA expression in NATs, and Grade I, Grade II, Grade III or Grade IV tumour samples. (B) Kaplan‐Meier survival analysis of overall glioma patients according to the difference of FBXL7 expression. (C) Kaplan‐Meier survival analysis of Grade IV glioma patients according to the difference of FBXL7 expression. (D) FBXL7 mRNA level was analysed by real‐time PCR in NHAs, A172, LN229, H4, U87 and U251 cells. (E) FBXL7 protein level was analysed by Western blotting in NHAs, A172, LN229, H4, U87 and U251 cells. *P < .05; **P < .01

The results of real‐time PCR assay in different grades of glioma tissues (Grades I, II, III and IV according to WHO) and adjacent normal brain tissues (NAT) to determine level of FBXL7 expression reveal significantly up‐regulated FBXL7 expression in glioma tissues all four grades compared with NAT specimens (Figure 1A), which improved progressively as the glioma grades increased (Figure 1A). To further assess this association, the glioma specimens were categorized into high and low FBXL7 groups with the cut‐off point being the mean of FBXL7 expression. Poor survival in cases with higher FBXL7 expression than those with lower levels in the glioma patients was observed on Kaplan‐Meier survival analysis (Figure 1B; *P* < .001). Furthermore, on analysing Grade IV of glioma samples, the cases with higher FBXL7 expression exhibited a shorter survival compared with those in low expression groups (Figure 1C; *P* < .001). In addition, glioma cells exhibited a remarkably up‐regulated FBXL7 level than that in NHAs (normal human astrocytes) (Figure 1D,E). The above data indicated that FBXL7 level was increased in glioma tissues and cells, and relative to with the grade and poor prognosis of glioma.

### 3.2 Knock‐down of FBXL7 in glioma cells impeded proliferation, migration and invasion and enhanced TMZ‐cytotoxicity

**Figure 2:**
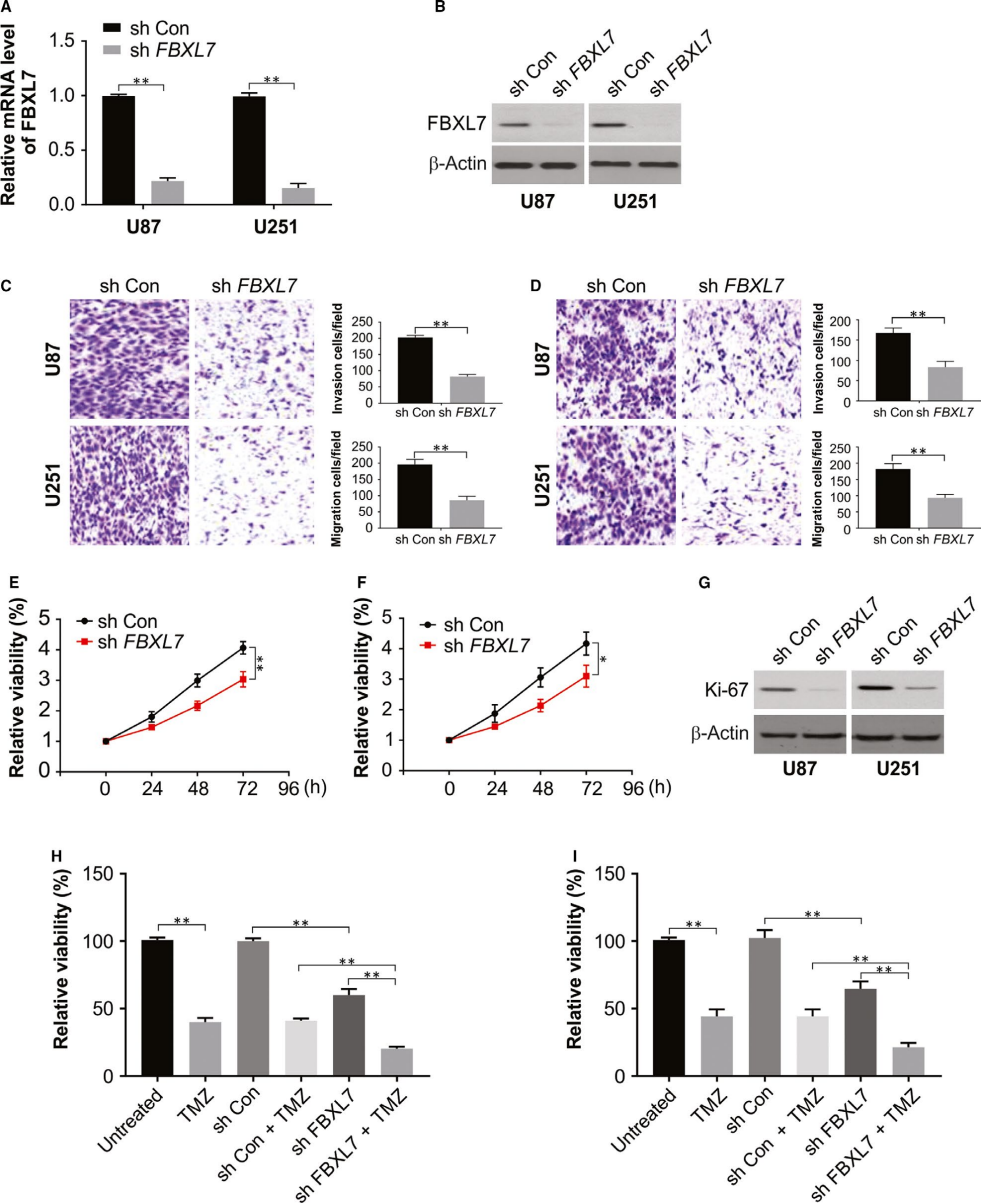
FBXL7 knock‐down suppressed invasion, migration, proliferation and potentiated TMZ sensitivity in glioma cells. (A) U87 and U251 cells were infected with sh con or sh *FBXL7* lentiviruses. FBXL7 mRNA level was examined by real‐time PCR at 24 hours after infection. (B) FBXL7 protein level was examined by western blotting at 24 hours after infection. (C and D) After 48 hours of infection, the effect of FBXL7 loss on glioma cell migratory and invasive abilities was assessed by Transwell migration and invasion assay. (E and F) At the indicated time‐points post‐infection, cell viability was analysed by CCK‐8 assay. (G) Ki‐67 protein level was analysed by Western blotting at 24 hours after infection. (H and I) U87 and U251 cells were infected with sh con or sh *FBXL7* lentiviruses. At 24 hours upon infection, infected or uninfected cells were stimulated with DMSO or TMZ (100 μmol/L) for another 48 hours. Then, cell viability was detected by CCK‐8 assay. Results were expressed as means ± SD of 3 independent experiments. **P < .01

Next, the mRNA and protein levels of FBXL7 were estimated through real‐time PCR and Western blotting experiments to be reduced significantly in sh *FBXL7* lentiviruse‐infected U87 and U251 cells than that in sh con lentiviruse‐infected cells (Figure 2A,B). Further, after the knock‐down of FBXL7, invasive and migratory capacities of U87 and U251 cells were markedly weakened (Figure 2C,D) In addition, an obviously down‐regulated cell proliferative ability was exhibited by U87 and U251 cells harbouring silenced FBXL7 (Figure 2E,F), which was confirmed by dramatic repression of cell proliferative marker Ki‐67 levels (Figure 2G). Cell viability was found to be reduced significantly in U87 and U251 cells stimulated with TMZ when compared to cells that were DMSO‐treated (control) (Figure 2H,I), and this inhibitory effect was further enhanced by FBXL7 knock‐down (Figure 2H,I), indicating that the loss of FBXL7 loss strengthened the cytotoxicity in glioma cells mediated by of TMZ.

### 3.3 FBXL7 is targeted by miR‐152‐5p

**Figure 3:**
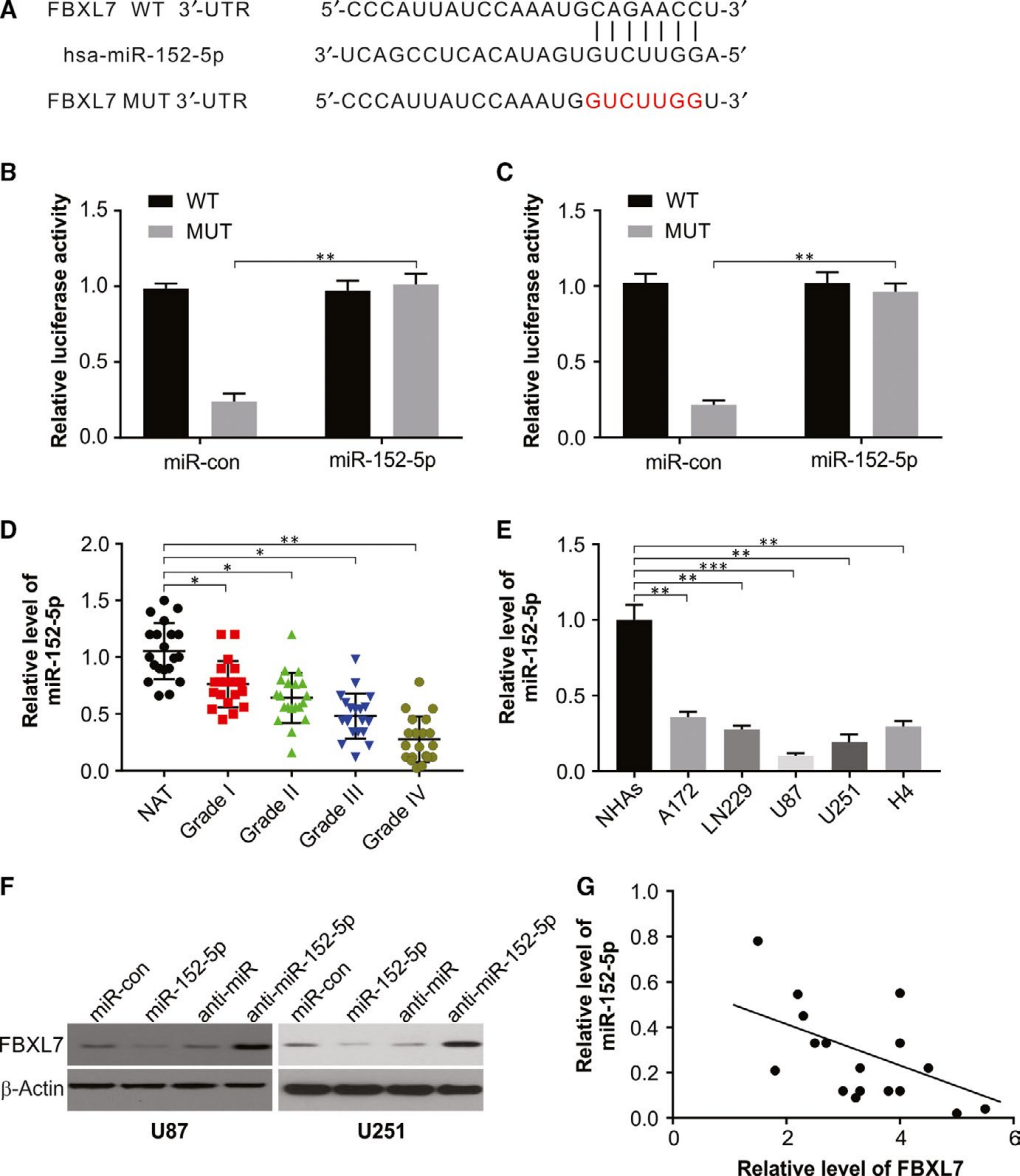
miR‐152‐5p targeting FBXL7 in glioma. (A) Putative complementary sites between miR‐152‐5p and FBXL7 3′‐UTR, and mutant sites in FBXL7 3′‐UTR‐MUT reporter. (B and C) U87 and U251 cells were cotransfected with FBXL7 3′‐UTR‐WT, FBXL7 3′‐UTR‐MUT reporter and miR‐152‐5p mimic or miR‐con, followed by the determination of luciferase activities at 48 hours post‐transfection. (D) miR‐152‐5p level was analysed by real‐time PCR assay at NATs, and Grade I, Grade II, Grade III or Grade IV glioma samples. (E) miR‐152‐5p level was analysed by real‐time PCR in NHAs and glioma cells. (F) The effect of miR‐152‐5p overexpression or loss on FBXL7 protein expression was measured by Western blotting at 24 hours after transfection in U87 and U251 cells. (G) Spearman's correlation analysis of FBXL7 and miR‐152‐5p level in Grade IV glioma tissues. Results were expressed as means ± SD of 3 independent experiments. *P < .05; **P < .01; and ***P < .001

The bioinformatics analysis of 3′‐UTR of FBXL7 mRNA through the website (http:// mirtarbase.mbc.nctu.edu.tw/php/search.php) which reveals matched seed sequence for miR‐152‐5p presents that (Figure 3A). Luciferase assay in cells U87 and U251 after overexpressing miR‐152‐5p revealed that the FBXL7 3′‐UTR‐WT reporter had remarkably repressed luciferase activity, but no such extensive effect was observed in cells harbouring FBXL7 3′‐UTR‐MUT reporter (Figure 3B,C), indicating a putative interaction between miR‐152‐5p and FBXL7 3′‐UTR via binding sites that were predicted. Additionally, with enhancement in tumour grade of glioma tissues, gradual down‐regulation of miR‐152‐5p was observed (Figure 3D). Besides, compared to NHAs, a significant inhibition of miR‐152‐5p was found in glioma cells (Figure 3E). Alternately, overexpressed miR‐152‐5p led to strikingly reduced FBXL7 expression in U87 and U251 cells, and significantly enhanced in cells depleted with miR‐152‐5p (Figure 3F), and this relationship was observed in glioma tissues of Grade IV (WHO) (Figure 3G). Thus, our findings indicated that FBXL7 is clearly a target of miR‐152‐5p in glioma.

### 3.4 Overexpression of miR‐152‐5p repressed migration, invasion and proliferation of glioma cell

**Figure 4:**
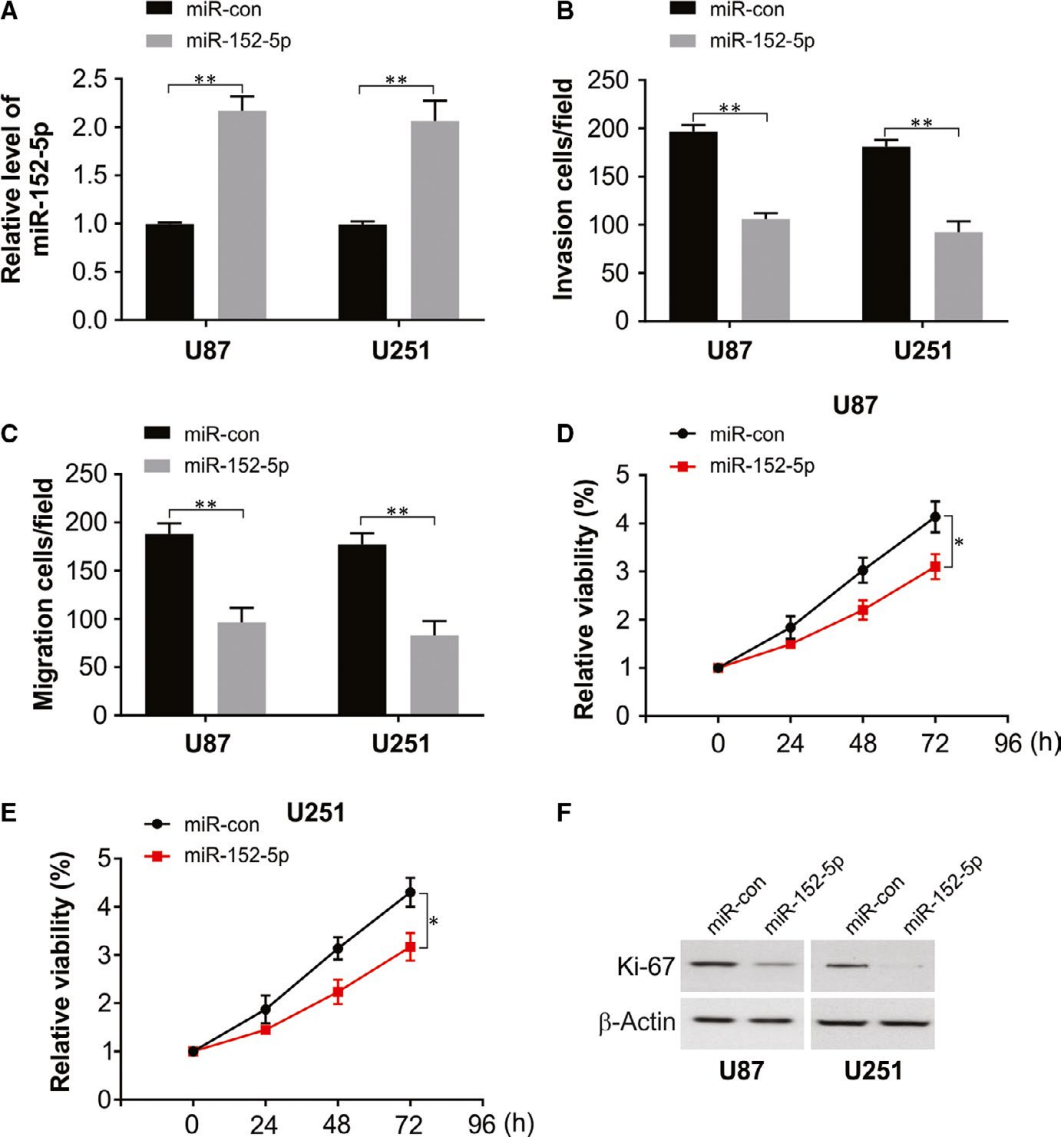
miR‐152‐5p overexpression inhibited glioma cell migration, invasion and proliferation. (A–F) U87 and U251 cells were transfected with miR‐152‐5p mimic or miR‐con. (A) miR‐152‐5p level was measured at 24 hours after transfection. (B and C) Cell migratory and invasive abilities were evaluated by Transwell migration and invasion assay at 48 hours following transfection. (D and E) The influence of miR‐152‐5p overexpression on cell viability was analysed by CCK‐8 assay. (F) Ki‐67 protein level was measured by Western blotting 24 hours later. *P < .05; **P < .01

Next, transfection of U87 and U251 cells was performed with a mimic of miR‐152‐5p which effectively elevated miR‐152‐5p level, as confirmed by real‐time PCR assay (Figure 4A). Sebsequent gain‐of‐function assays revealed that overexpression of miR‐152‐5p triggered significantly downregulation of cell invasion (Figure 4B), migraion (Figure 4C) and proliferative (Figure 4D–F) capacities in U87 and U251 cells.

### 3.5 MiR‐152‐5p overexpression enhanced TMZ sensitivity in glioma cells by targeting FBXL7

**Figure 5:**
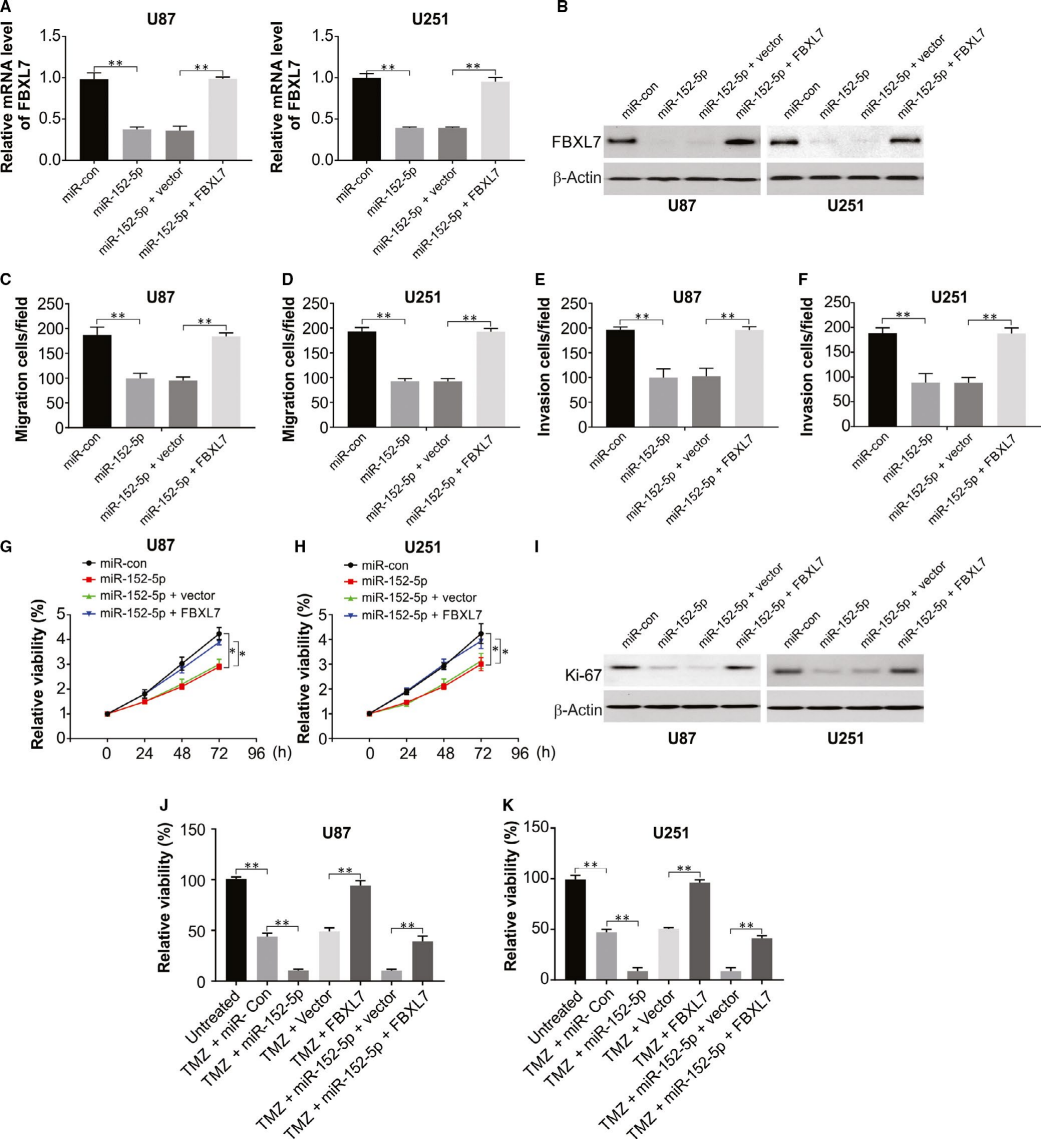
miR‐152‐5p overexpression suppressed migration, invasion, proliferation and enhanced TMZ‐induced cytotoxicity by targeting FBXL7 in glioma cells. (A–G) U87 and U251 cells were transfected with miR‐con, miR‐152‐5p mimic, miR‐152‐5p mimic + pcDNA3.1 empty vector or miR‐152‐5p mimic + pcDNA‐FBXL7, followed by the measurement of FBXL7 mRNA level (A), FBXL7 protein level (B), cell migratory (C and D), invasive (E and F) and viability (G and H) capacities, and Ki‐67 protein level (I). (J and K) U87 and U251 cells were transfected with miR‐con, miR‐152‐5p mimic, pcDNA3.1 empty vector, pcDNA‐FBXL7, miR‐152‐5p mimic + pcDNA3.1 empty vector or miR‐152‐5p mimic + pcDNA‐FBXL7 for 24 hours and then treated with TMZ (100μM) for another 48 hours. Cell viability was estimated by CCK‐8 assay. Results were expressed as means ± SD of 3 independent experiments. *P < .05; and **P < .01

Subsequently, the overexpression of FBXL7 in U87 and U251 cells could abolish miR‐152‐5p–mediated repression of FBXL7, as confirmed by Western blotting and real‐time PCR (Figure 5A,B). Restoration experiments improved the U87 and U251 cell migratory, invasive (Figure 5C–F) and proliferative (Figure 5G–I) abilities in miR‐152‐5p–enforced cells following an increase in levels of FBXL7, which also enhanced TMZ‐mediated inhibition of U87 and U251 cell viability. FBXL7 overexpression hindered miR‐152‐5p‐induced cell viability downregulation in TMZ‐stimulated U87 and U251 cells (Figure 5J,K). Thus, these data unveiled that miR‐152‐5p suppressed migration, invasion, proliferation and enhanced TMZ‐induced cytotoxicity by targeting FBXL7 in glioma cells.

### 3.6 miR‐152‐5p overexpression or FBXL7 knock‐down in U87 cell‐derived models of glioma xenograft improved anti‐tumour effect mediated by TMZ and inhibited tumour growth

**Figure 6:**
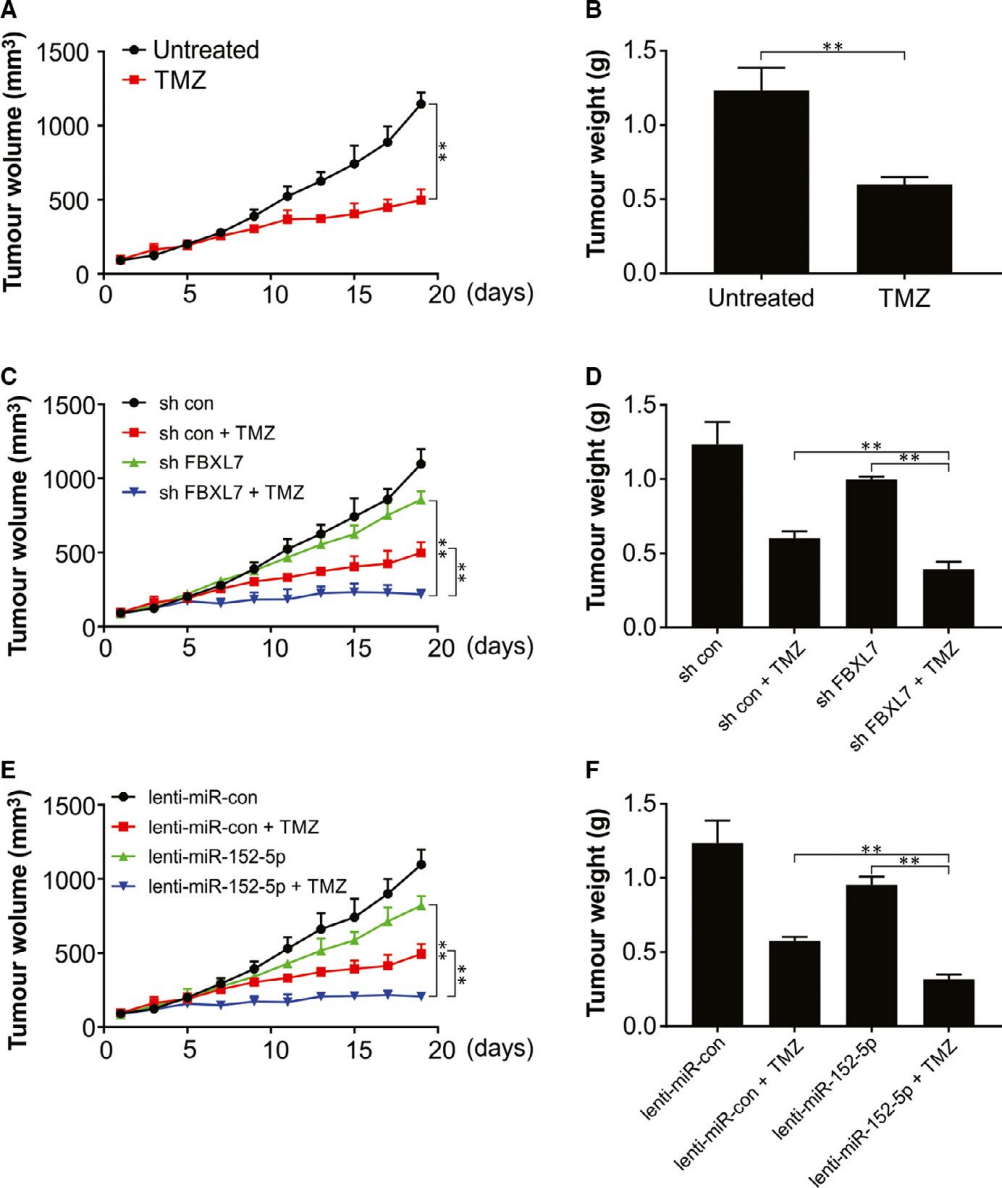
FBXL7 knock‐down or miR‐152‐5p overexpression inhibited tumour growth and enhanced TMZ‐mediated anti‐tumour effect in U87 cell‐derived glioma xenograft models. (A‐F) Uninfected U87 cells or U87 cells infected with sh con, sh *FBXL7*, lenti‐miR‐con or lenti‐miR‐152‐5p lentiviruses were injected subcutaneously into the right hind limb of mice. One week later, TMZ was administered intraperitoneally into mice daily at a dose of 20 mg/kg bodyweight for a total of 10 days. (A, C and E) Tumour volume was measured every 2 days using a caliper. (B, D and F) At the end of experiments, tumours were excised and weighed. **P < .01

We further observed that the silencing of FBXL7, TMZ stimulation alone, or overexpression of miR‐152‐5p led to reduced volume and weight of glioma xenograft tumour (Figure 6A–F). Depletion of FBXL7 or overexpression miR‐152‐5p in U87 cell‐derived glioma xenograft models also enhanced anti‐tumour effect mediated by TMZ (Figure 6A–F).

## 4 DISCUSSION

Gliomas are characterized as anaplastic astrocytomas, well‐differentiated low‐grade astrocytomas, and glioblastoma multiforme (GBM), a highly aggressive and deadly brain tumour in adults.^30^, ^31^ The treatment of GBM using post‐surgical chemotherapeutic agents and radiotherapy provides limited relief and the median survival of less than 1 year in GBM patients.^6^, ^32^, ^33^ FBXL7 is evolutionally highly conserved protein with a sequence identity of 98% between mouse and humans, and >95% in other species.^34^ However, in terms of the biological and regulatory role in cells, the characterization of FBXL7 remains relatively poor.^16^, ^35^ Extensive investigation is required to investigate whether FbxL7 is a part of a complex that initiates mitotic arrest causing cell death, or it acts as an oncoprotein. In the current study, we observed markedly up‐regulated expression of FBXL7 glioma cells, in accordance with earlier reports.^16^, ^36^ Further, FBXL7 expression correlated with the grade of glioma and poor prognosis.^15^ In vitro functional analysis showed that knock‐down of FBXL7 repressed invasion, proliferation and migration of glioma cell and impeded in vivo growth of glioma xenograft tumour. We could also show that knock‐down of FBXL7 improved inhibitory effects mediated by TMZ glioma xenograft growth in vivo and on viability of glioma cell in vitro and that FBXL7 expression was inhibited in glioma cells by miR‐152‐5p via direct interaction. Our findings further reveal gradual repression of miR‐152‐5p level with the increase in the grade of glioma. In contrast, induced expression of FBXL7, indicating the effect of endogenous miR‐152‐5p on FBXL7 expression.

In several cancers, much focus has been paid on the examining molecular biomarkers carrying vital information in glioma prevention, diagnosis and prognosis, as well as treatment.^37^ Knock‐down of a commonly reported oncogenic factor, FBXL7 impeded in vitro migration and invasion, as well as the epithelial‐mesenchymal transition in gastric cancer cells and stalled metastasis of gastric cancer cells in vivo, reduced invasive abilities and initiated cell apoptosis and cell cycle arrest in tongue squamous cell cancer.^35^

Abnormal expression of miR‐152 was observed for the first time in mouse colon in 2002, and there are increasing proofs to indicate the tumour‐suppressive effect of miR‐152, associated with human cancer cell migration, invasion and proliferation.^38^, ^39^, ^40^ The miR‐152 gene is located at 17q21.32 in humans.^41^ After processing, the pre–miR‐152 (precursor miR‐152) is moved to the cytoplasm and forms a miR‐152 duplex after being cleaved by the Dicer giving rise to two mature miRNAs of different lengths and sequences, miR‐152‐3p and miR‐152‐5p (miR‐152*), of which miR‐152‐3p has a higher frequency of occurrence in each species than miR‐152‐5p.^41^ So far, miR‐152‐3p has been implicated in regulating the progression and development of several types of cancers such as gastric carcinoma.^39^

For the treatment of glioma, standard post‐resection chemotherapy is given, which includes TMZ, although because of therapeutic resistance and tumour recurrence, there is often no decline in rate of mortality.^42^, ^43^ TMZ is an agent for alkylating, that causes DNA methylation at the O^6^ position of guanine causing incorrect pairing of thymine with O^6^‐methylguanine, which initiates the mismatch repair system, causing a double‐strand break in the genome and subsequent cell cycle arrest and apoptosis.^8^ TMZ resistance is induced when O^6^‐methylguanine‐DNA methyltransferase (MGMT) demethylates the O^6^ position of guanine.^44^ Hence, in patients with silenced MGMT by methylated MGMT promoter showed better 2‐year and median survival after receiving TMZ chemotherapy and radiotherapy in combination.^45^ By depleting MGMT, MGMT pseudo substrates were expected to remove resistance in patients with GBM that was TMZ‐resistant; however, no significant TMZ sensitivity restoration was seen in associated clinical trials. Therefore, there is a need for developing new approaches to make patients more sensitive to efficient TMZ chemotherapy.^45^, ^46^

We could show here after miR‐152‐5p overexpression, the suppression of glioma cell migration, proliferation and invasion, as well as enhanced in vitro TMZ‐triggered decline in glioma cell viability, and these effects were nullified by enhanced FBXL7. In vivo, miR‐152‐5p overexpressed in mouse xenograft models for glioma could inhibit the growth of the tumour and improved the anti‐tumour effect mediated by TMZ. Alternately, miR‐152‐5p expression could be induced by TMZ in glioma cells. This led us to hypothesize that the anti‐tumour effects of TMZ might be by regulation of miR‐152‐5p/FBXL7 axis in glioma.

However, current research has some limitations. First, epithelial‐mesenchymal transition (EMT) is related to initiation of metastasis in cancer progression. In the current study, we detected migration and invasion in sh *FBXL7* cells. The effects of miR‐152‐5p/FBXL7 on EMT will be checked in the subsequent research. Second, in the xenograft mouse model, IHC/IF staining of Ki‐67, miR‐152‐5p levels and FBXL7 protein levels should be detected in the tumours. In addition, how FBXL7 regulates glioma function will be investigated in the future studies.

Taken together, we demonstrate here for the first time a regulatory axis miR‐152‐5p/FBXL7 in glioma tumorigenesis and indicate that FBXL7 and miR‐152‐5p may be a potential treatment target for glioma, singly or combined with TMZ. Also, levels of FBXL7 and miR‐152‐5p can act as the secondary prognostic indicators with increased miR‐152‐5p and reduced FBXL7 levels indication better prognosis. However, the downstream or upstream regulatory molecules or pathways in gliomas must be explored to assess the molecular basis of the biomarker capacity of FBXL7. Also, the combined effect of TMZ, miR‐152‐5p, and FBXL7 on xenograft growth, glioma cell migration, proliferation, invasion and TMZ resistance must be investigated.

## CONFLICT OF INTEREST

There is no conflict of interest.

## AUTHOR CONTRIBUTIONS

SK, YF, BW, YC, RH and ZZ performed the experiments. SK and ZZ designed the study. SK and YF analysed the data. SK and ZZ wrote the draft. All authors reviewed and approved the manuscript.

## Data Availability

The data used to support the findings of this study are available from the corresponding author upon request.
